# The ‘Save the Meniscus’ philosophy and the ‘Iceberg’ concept

**DOI:** 10.1002/jeo2.70596

**Published:** 2025-12-28

**Authors:** Angelo V. Vasiliadis, Vasileios Giovanoulis, Theodorakys Marín Fermín, Luca Macchiarola, Nicholas Colyvas

**Affiliations:** ^1^ Department of Orthopaedic Surgery St. Luke's Hospital Panorama, Thessaloniki Greece; ^2^ Department of Orthopaedic Surgery Croix‐Rousse Hospital Lyon France; ^3^ Orthopedic Department Centre Hospitalier de Versailles—Hopital Andre Mignot Le Chesnay‐Rocquencourt France; ^4^ Clínica Santa Sofía Av. Principal de Santa Sofía El Cafetal, Caracas Venezuela; ^5^ Department of Orthopedics and Trauma Surgery Fondazione Casa Sollievo Della Sofferenza IRCCS San Giovanni Rotondo Italy; ^6^ Department of Orthopaedic Surgery University of California‐San Francisco San Francisco California USA

**Keywords:** meniscal repair, meniscectomy, meniscus, posterior root, save meniscus

**Figure 1:**
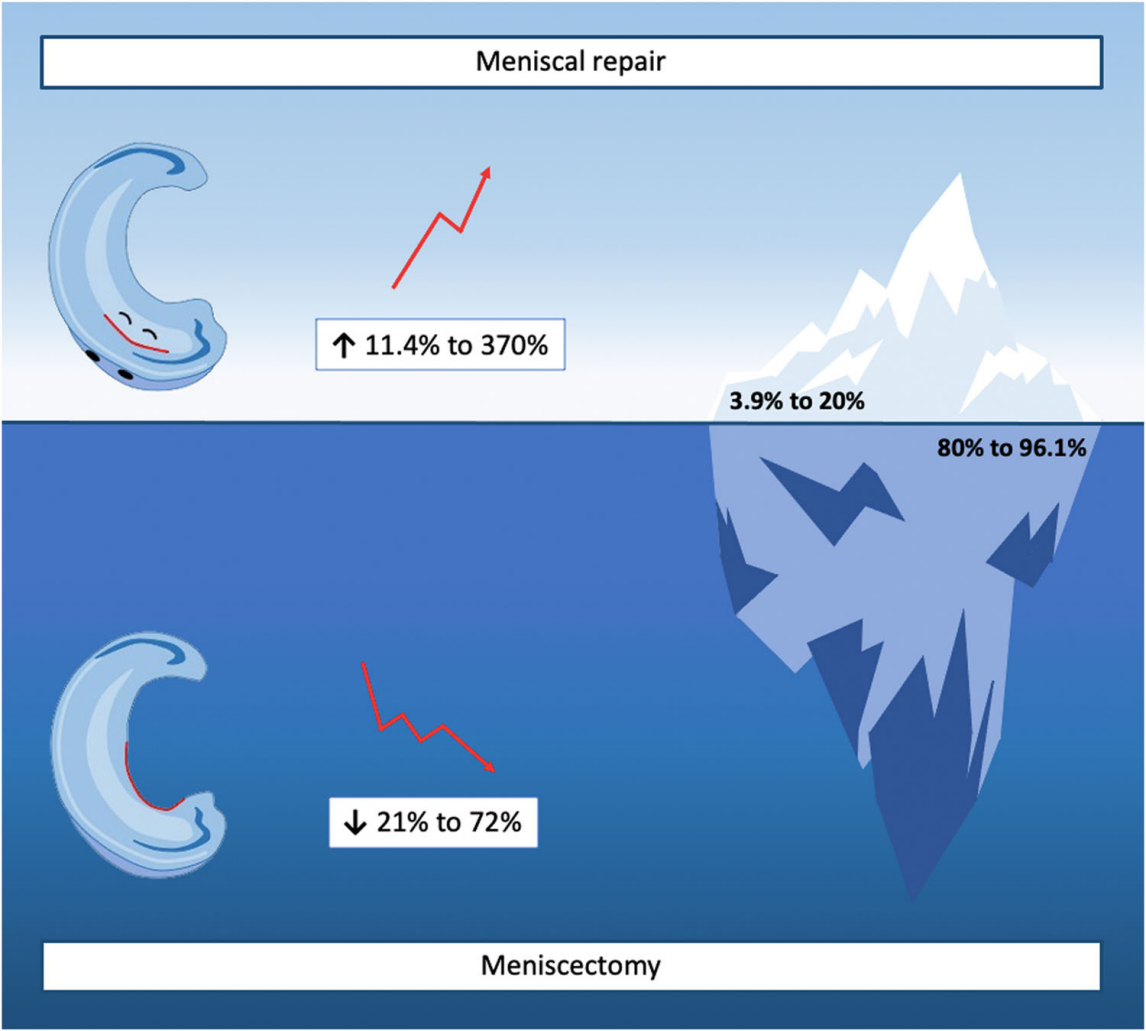
The ‘Iceberg’ concept in meniscal surgery. Over the past two decades, meniscal repair has increased by 11.4%–370%, while meniscectomy has declined by 21%–72%. Despite this shift, meniscal repair accounts for only 3.9%–20% of procedures, with meniscectomy still comprising 80%–96.1%.

In recent years, there has been growing interest in preservation‐focused approach to the management of meniscal tears. An increasing number of surgeons now advocate for the ‘Save the Meniscus’ philosophy, expanding the envelope of what is considered repairable [1, 2, 3]. While it is acknowledged that not every tear is amenable to repair, and not all surgeons possess the same level of expertise, ongoing education and skill development can enable surgeons to turn seemingly unrepairable tears into viable repair candidates. In this editorial, we support this evolving mindset and introduce the ‘Iceberg’ concept in the surgical management of meniscal tears (Figure 1), a metaphor that illustrates how the visible, commonly addressed cases represent only a small portion of the true repairable potential.

The meniscus acts as a vital anatomical part of the knee joint and plays a fundamental role in its function. It helps distribute loads, absorb forces, stabilize the joint, facilitate lubrication and contribute to the nutrition of the knee joint [2]. Traditionally, meniscectomy (partial or total) was routinely performed as the gold standard treatment to alleviate symptoms in the short term. However, it is now well known that the absence or tear of the meniscus has dramatic and irreversible consequences for the joint cartilage in the long term, including the early onset of osteoarthritis [2, 4, 5]. Since LaPrade first introduced the principle of ‘Save the Meniscus’ in 2007 [6], and it was later reinforced by Lubowitz in 2011 [7], there has been a significant shift in the surgical management of meniscal tears. In this regard, a majority of orthopaedic surgeons feel that the future of meniscus management should focus on meniscal preservation principles [8].

Today, the surgical treatment of meniscal tears has evolved, with a significant increase in arthroscopic meniscal repair and a substantial reduction in partial meniscectomy (Figure 1) [4, 8, 9, 10, 11]. Meniscal repair has become one of the fastest‐growing areas in sports medicine, where the global proportion of arthroscopic meniscal repair is increasing by 11.4% to as much as 370% over the last two decades [4, 8, 9, 10, 11, 12, 13, 14]. In contrast, arthroscopic meniscectomy has shown a global reduction by 21%–72% over the same study period [4, 8, 9, 10, 11]. Interestingly, studies from Brazil and Korea have presented increases in both meniscectomy and meniscal repair, with the proportion of meniscal repair being higher [13, 14]. This confirms that while meniscal surgical volume may be increasing overall, there is a growing preference for meniscal repair when possible. These findings also highlight that meniscal surgeries remain among the most commonly performed procedures in orthopaedics. Interestingly, two large‐scale studies from the United States [10, 12], covering the period from 2005 to 2020 and analysing a combined total of 2,465,357 meniscus surgeries, revealed different trends in meniscal management. Abrams et al. (2005–2011) reported an increase in both meniscectomies (4.7%) and meniscus repairs (11.4%), with the latter being statistically significant [12]. In contrast, a more recent study by Bergstein et al. (2010–2020) showed a significant decline in meniscectomies (53%) alongside a substantial increase in meniscal repairs (40%) [10]. Overall, these findings underscore the variability in surgical practices across different parts of the world.

Despite the encouraging trend in the global orthopaedic community regarding the overall increase in meniscal repair rates, the bulk of the iceberg still seems to be ‘submerged’ (Figure 1). Currently, the global rate of meniscal repair is often reported as a range between 3.9% and 20% of all arthroscopic meniscal procedures [4, 8, 9, 10, 11, 12, 13, 14]. Arthroscopic meniscectomy remains far more common, accounting for 80%–96.1% of arthroscopic meniscal procedures [4, 8, 9, 10, 11, 12, 13, 14]. The Iceberg concept effectively illustrates the surgical management of meniscal tears. The small and visible tip of the iceberg represents meniscal repair, which is a less common and more demanding procedure. In contrast, the much larger, hidden portion symbolizes the arthroscopic meniscectomy, which remains the more frequently performed meniscal procedure. This visually highlights that despite advancements in surgical techniques and surgeons' willingness to preserve the meniscus, repair rates remain relatively low.

The principle ‘Save the Meniscus’ is more important than ever if we want to upend the Iceberg. We believe this remains true despite the potential risks of failure, the higher cost of repair, reoperation rates and the demands of lengthy and restrictive rehabilitation protocols associated with meniscal repair [1, 3]. Modern advances in arthroscopic techniques have made the meniscal repair a more reproducible and reliable procedure for surgeons. Although there has been a significant increase in the amount of meniscal repair procedures in recent years [15] and progress in meniscal preservation is encouraging, we highlight that there remains quite a way to go before we can abandon the Iceberg for a better‐balanced metaphor of meniscus surgery. Looking ahead, we hope that the continued focus on appropriate indications and surgical techniques for meniscus repair moves us in this direction.
